# Monthly Summary

**Published:** 1886-04

**Authors:** 


### Monthly Summary.

PREVENTION OF BAD TEETH.—An exchange says the troubles which arise from disease of the teeth, or from their loss, are not always directly referable to their cause. When actual toothache is present there is of course little doubt, and the remedy of extraction at once presents itself. At the same time, it must be remembered that the forceps does not undo the work of disease or make amends for its ravages. A jaw which has lost the best part of its function with its teeth, or which bites unequally with the scattered survivors of its former armament, is but a deceitful guardian of the passage of the stomach; while it seems to do duty in mastication, it passes intact much that is unfit for the immediate action of the gastric juice. Were the relationship between bad teeth and dyspepsia, with its consequent discomforts, better understood, we should probably hear less of the prevalence of dental caries. Greater attention would then be paid to the small organs whose obscure influence on the general health so fully justifies their preservation. Specific constitutional disease, drugs, and other special factors no doubt account for a certain amount of dental decay. Neglect, however, accounts for much more. Want of care in choosing food, and particularly in cleansing the teeth, has nearly everything to do with the dental worries of a great many people. A point well worthy of note in this connection is that most of the pernament set of teeth come into active operation during childhood or early youth. It is hardly to be expected that children, if let alone, will pay much attention to the state of their mouths, unless driven to notice an aching stump. Thus it happens that most children have lost one or several teeth before they are well into their teens. Here, then, there is need for parental supervision. Mr. W. M. Fisher, of Dundee, has been led by certain observations, which proved the defective condition of the teeth in a majority of school children, to suggest that some regular system of supervision by a dentist be adopted as a part of school management. The expenses he would have defrayed by the parents, or, should they be too poor, by the State, out of the education grant. This plan has been actually adopted in the parochial school at Anerly, in Surrey. We have long been of opinion that it would be most desirable if the health of children in all schools could by some plan be periodically passed under review by a medical inspector. The possible obstacle to such an arrangement would be the expense. This may not prove insuperable, and if it does not, we may hope that this method, and also some plan of dental supervision, may find their place among the recognized forms of school discipline.—*Dental Register.*

---

PROSECUTION OF A DENTIST.—At the Manchester Assizes last month, before Mr. Justice Day, an action was brought against Mr. James Jackson, dentist, Burnley, in which the plaintiff, Mr. Robert Jackson, farmer, sought to recover damages for the alleged seduction of his daughter whilst under the influence of nitrous oxide. There was also a cross action for slander brought against the plaintiff. The trial occupied nearly three days-

His Lordship, in summing up, said the one substantial issue for the jury was, did James Jackson, the dentist, or did he not, administer gas or some narcotic to the young woman, Margaret Ann Jackson, and did he, while she was under the influence of some anæsthetic, crimminally assault her? That was the question they had to determine, and it was a question of the very gravest moment. The consequences to the one side or the other must necessarily be of the most serious character. The charge which was made against the dentist was one of assault under circumstances of the most aggravated and and nefarious nature. The charge, on the other hand, of which the woman would be guilty, if she had made a false accusation, was one of the most wicked, odious, and vile that could be brought by one human being against another. The case was one of a most extraordinary character, and one which, he was happy to think, was very rarely raised in a court of justice. It was one which demanded at the hands of the jury, as he knew it would most assuredly receive, their deepest and most anxious attention, so that to the utmost of their ability they might do justice between the parties. He did not hesitate to say that the question was of an extremely difficult character; but it was one which he was confident the jury would, using their own good sense, solve to their thorough satisfaction; and if they did solve it to their satisfaction it should be satisfactory to all well-minded people. He would say nothing about damages, because it was unnecessary. The parties probably were none of them in position to pay damages. That, however, was utterly unimportant, and should not affect the amount of damages. It was unnecessary for him to say a word about damages, because he should not venture to put any limit upon the damages which they might award to either one side or the other.

The jury retired to consult on the case, and after deliberating for three hours, returned to court and stated that there was no possibility of their coming to an agreement. The Judge thereupon discharged them.—*Dental Record.*

---

THE FLUCTUATIONS IN COCAINE.—Notwithstanding the fact that the consumption of cocaine is steadily on the increase, and that remarkable progress has been made in its introduction as a local anæsthetic, prices having declined just as rapidly and even now there is a perceptible weakness about the market which forebodes lower values. The precipitation of values is owing principally to competition in its manufacture and the decline in the price of leaves. The discovery of cocaine is scarcely two years old and the furore created at the time has spread to the uttermost parts of the earth. It had many enemies, like other new articles, but no remedial agent ever receivd so much abuse as cocaine, which has fortunately reacted on its defamers to the added popularity of this drug. When first brought on this market it sold for two dollars and a half per drachm, then advanced to ten dollars, and the free advertising it received through the medical press attracted the attention of American chemical manufacturers who were not slow in appreciating its importance in the medical world. One after another started to produce cocaine muriate and the field is now occupied by five manufacturers. The largely increased production tended to cheapen the article, and this had the natural effect of increasing its consumption, although the merits of cocaine are alone sufficient to make its use universal, but more sparingly, if the high figures ruling at the start had continued. The market at present is quoted at eleven to twelve dollars per ounce for bulk.

Manufacturers are surprised at the success of their venture. They characterize its sale as being something "wonderful" and the movement has kept up unchecked, with the prospects promising of continuing so.—*Drug Reporter.*

---

MR. JONATHAN HUTCHINSON believes that there is no reason to think that the transmission of syphilis is ever a thing of less or more, but rather that if a child inherits any taint it inherits the whole malady; the varying degrees of severity are to be explained in the same way as we explain the differences observed when scarlet fever is passing through a community. He adduces the hypothesis of M. Parrot that rickets is due to syphilis, and sees no reason why syphilis and rickets should not mix. The question as to whether deep ulcerations of palate and pharynx, when met with in young persons, are usually due to syphilis or to scrofula, evidence pointed strongly to the conclusion, in Mr. Hutchinson's opinion, that syphilis was chiefly responsible for such ulcerations.—*Dental Advertiser.*

